# Cucumber *glossy fruit 1* (*CsGLF1*) encodes the zinc finger protein 6 that regulates fruit glossiness by enhancing cuticular wax biosynthesis

**DOI:** 10.1093/hr/uhac237

**Published:** 2022-02-21

**Authors:** Yan Yang, Congxi Cai, Yipeng Wang, Yanran Wang, Haolun Ju, Xuehao Chen

**Affiliations:** School of Horticulture and Plant Protection, Yangzhou University, Yangzhou, 225009, Jiangsu Province, China; School of Horticulture and Plant Protection, Yangzhou University, Yangzhou, 225009, Jiangsu Province, China; School of Horticulture and Plant Protection, Yangzhou University, Yangzhou, 225009, Jiangsu Province, China; School of Horticulture and Plant Protection, Yangzhou University, Yangzhou, 225009, Jiangsu Province, China; School of Horticulture and Plant Protection, Yangzhou University, Yangzhou, 225009, Jiangsu Province, China; School of Horticulture and Plant Protection, Yangzhou University, Yangzhou, 225009, Jiangsu Province, China

## Abstract

Cucumber glossiness is an important visual quality trait that affects consumer choice. Accumulating evidence suggests that glossy trait is associated with cuticular wax accumulation. However, the molecular genetic mechanism controlling cucumber glossiness remains largely unknown. Here, we report the map-based cloning and functional characterization of *CsGLF1*, a locus that determines the glossy trait in cucumber. *CsGLF1* encodes a homolog of the Cys_2_His_2_-like fold group (C2H2) -type zinc finger protein 6 (ZFP6) and its deletion leads to glossier pericarp and decreased cuticular wax accumulation. Consistently, transcriptomic analysis demonstrated that a group of wax biosynthetic genes were downregulated when *CsZFP6* was absent. Further, transient expression assay revealed that CsZFP6 acted as a transcription activator of cuticular wax biosynthetic genes. Taken together, our findings demonstrated a novel regulator of fruit glossiness, which will provide new insights into regulatory mechanism of fruit glossiness in cucumber.

## Introduction

Cucumber (*Cucumis sativus*), which belongs to the family of *Cucurbitaceae*, is widely cultivated in the world [1]. According to FAOSTAT 2020 (https://www.fao.org/faostat), the global cucumber production has been estimated to be close to 91 million tons. Fruit glossiness is a commercially important quality trait. Notably, compared with dull fruits, glossy cucumber fruits are more appealing to consumers [2, 3].

Accumulating evidence suggests that glossy trait is related to cuticular wax [4–8], an outermost hydrophobic barrier covering the outmost surface of terrestrial plants [9]. Cuticular wax is a complex mixture of very long chain fatty acids (VLCFAs; C20 to C34) and their derivatives synthesized by epidermal cells [10, 11]. The physical and chemical properties of cuticular waxes determine pivotal functions of plants. Indeed, besides influencing glossiness, waxes play important roles in limiting uncontrolled nonstomatal water loss [9, 12], protecting plants against UV radiation [13], and defense against pathogens [11]. Over the past three decades, forward genetic experiments in *Arabidopsis* have provided significant advances in our understanding of wax biosynthesis. C16 and C18 fatty acids were reported to be precursors of VLCFA biosynthesis, which are catalyzed by the enzymes of the endoplasmic reticulum (ER)-associated fatty acid elongase (FAE) complexes [14]. Wax aliphatic components are then produced via two distinct pathways: (i) the alcohol-forming pathway, which yields primary alcohols and wax esters; and (ii) the alkane-forming pathway leading to the formation of aldehydes, alkanes, secondary alcohols, and ketones [10]. With the help of wax-deficient mutants, numerous enzymes associated with the alcohol- and the alkane-forming pathways have been identified, which comprise the FAE-related enzymes including 3-ketoacyl-CoA synthetases (KCSs), 3-ketoacyl-CoA reductases (KCRs), and enzymes participating in the modification of VLCFA, such as ECERIFERUM1 (CER1), ECERIFERUM3 (CER3), and MID-CHAIN ALKANE HYDROXYLASE 1 (MAH1) [15–19]. In cucumber, both CsCER1 and CsCER3 have been demonstrated to be involved in wax biosynthesis [20, 21].

While wax biosynthetic pathway has been well elucidated, limited transcription factors have been shown to be involved in the regulation of cuticular wax formation and glossiness in cucumber. In this study, we identified *CsGLF1*, a locus controlling fruit glossiness. *CsGLF1* encodes the CsZFP6 transcription factor, and its mutation conferred cucumber glossiness by suppressing cuticular wax biosynthesis. Our findings identified a novel regulator of cucumber fruit glossiness and will provide new insights into the genetic mechanism of fruit glossiness.

## Results

### Preliminary mapping of the *CsGLF1* locus

**Figure 1:**
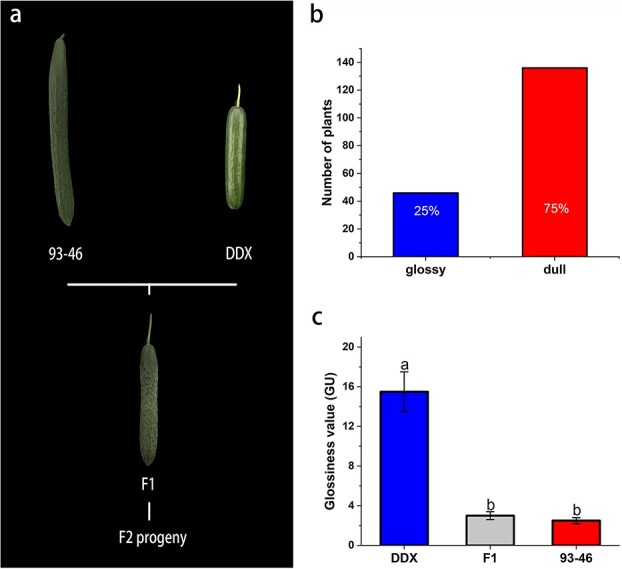
Phenotypic differences in cucumber glossiness. **a** Glossiness assessment of 7-dpp fruits from two parental lines and their F_1_. The glossy parental line DDX was crossed with the dull parental line 93–46 and resulting F_1_ hybrid plants displayed dull fruits. **b** Screening of F_2_ progeny for glossy fruits (approximately 25% of the plants) and dull fruits (approximately 75% of the plants). Fruits for phenotypic observation were harvested at 7 d post-pollination (dpp). **c** Gloss unit value of 7-dpp fruits from two parental lines and their F_1_. The error bars indicate the standard errors of five replicates. Different letters indicate significant differences according to the Tukey test (*P* < 0.05).

Previous findings indicated glossy trait is controlled by a single recessive gene *d* [3, 22]. However, when we crossed DDX, a glossy European greenhouse-type inbred line, with a glossy north China-type inbred line JY35 to produce F_1_ hybrid, all obtained F_1_ individuals displayed dull appearance (Fig. S1, see online supplementary material), indicating that this trait is controlled by more than one gene. To gain better understanding of the underlying mechanism, recombinant inbred lines (RILs) derived from DDX and JY35 were generated. Then, a new F_2_ population was established by crossing a dull RIL 93–46 with DDX (Fig. 1a). The F_1_ hybrid plant derived from the cross between DDX and 93–46, which exhibited a dull appearance, was then selfed. The resulting F_2_ segregating population (182 plants) was analysed for the glossy phenotype. As shown in Fig. 1b, F_2_ plants approximately segregated as 3:1 (136 dull plants:46 glossy plants; χ2 = 0.0563 < χ2_0.05,1_ = 3.84) according to a χ^2^ test, implying that the glossy trait in DDX is controlled by a single recessive gene, designated as *Glossy Fruit 1* (*CsGLF1*) (Fig. 1b; Table S1, see online supplementary material). Gloss levels were also examined by a curve glossmeter, which verified that DDX fruit surfaces were glossier than those of the 93–46 and F_1_ (Fig. 1c).

**Figure 2:**
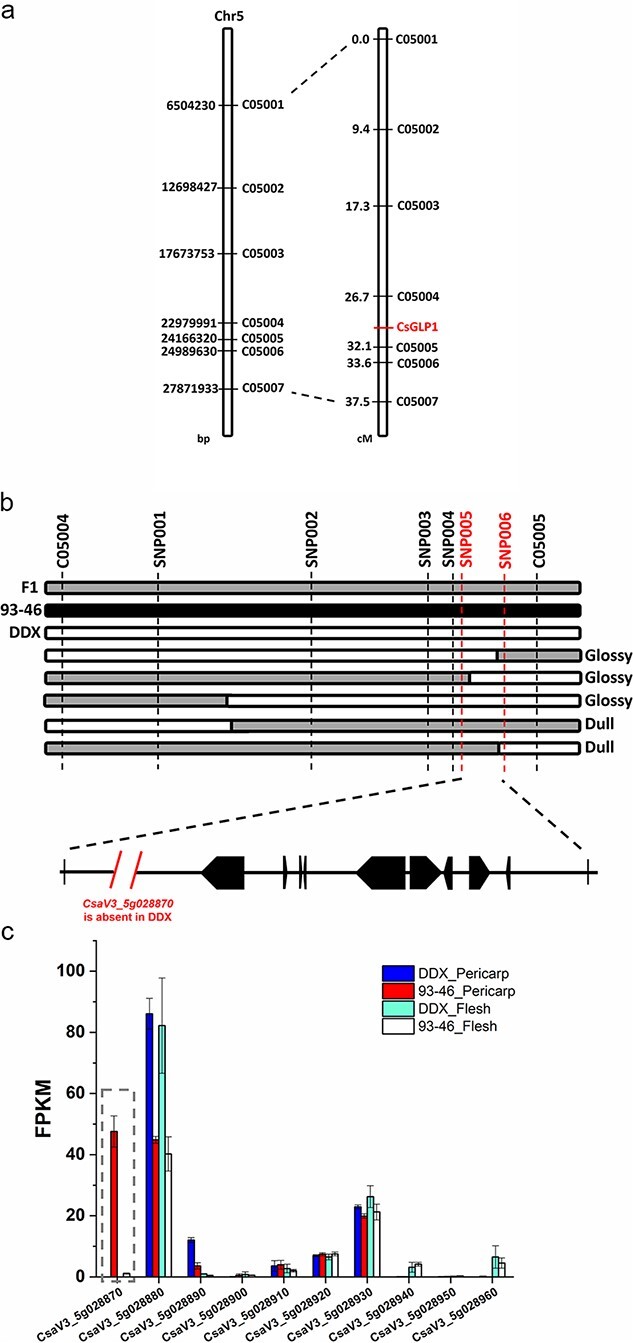
Mapping of the *CsGLF1* locus*.***a** Genetic map of *CsGLF1* (right, unit: cM) and the corresponding physical map (left, unit: bp). **b** Fine mapping of *CsGLF1*. Positional cloning delimited *CsGLF1* within the DNA segment between markers C05004 and C05005. Using recombinants, *CsGLF1* locus was further narrowed to a 111.6 kb genomic region containing 10 annotated genes according to Chinese Long cucumber v3 reference genome. A 4895 bp deletion in DDX leads to the complete loss of *CsaV3_5g028870*. Open, grey and filled bars represent homozygous fragments from DDX, possible crossover intervals and homozygous fragments from 93–46, respectively. Phenotype of recombinants is shown on the right. Black arrows indicate genes in the interval. **c** Expression levels of the 10 genes within the 111.6 kb region in 7-dpp fruits from the DDX and 93–46. FPKM, fragments per kilobase of transcript per million mapped reads. The error bars indicate the standard errors of three replicates.

Next, a total of 141 insertions/deletions (InDel) primers evenly distributed on seven chromosomes were designed to screen the parental lines based on resequencing data, of which 76 markers were polymorphic between the two parents (Table S2, see online supplementary material). Then, these 76 InDel markers were used to detect polymorphisms between two DNA pools (the glossy pool and the dull pool), and C05004 (Chr5: 22979991), C05005 (Chr5: 24166320), and C05006 (Chr5: 24989630) were identified polymorphisms between the two pools. Subsequently, these three markers together with their nearby InDel markers were applied to all F_2_ individuals to validate the linkage, identify recombinants and narrow the candidate mapping region. As a result, the *CsGLF1* locus was preliminarily mapped to an interval flanked by C05004 and C05005 on chromosome 5 (Fig. 2a).

### Fine-mapping and cloning of *CsGLF1*

To further narrow down the candidate region, two flanking markers C05004 and C05005 were used to genotype an expanded F_2_ population. Besides, six single nucleotide polymorphism (SNP) markers in the region of interest were developed and used to identify more recombinant individuals. Eventually, based on the genotypes and phenotypes of 17 F_2_ recombinants, the *CsGLF1* locus was delimited within a 111.6 kb genomic region between markers SNP005 and SNP006 (Fig. 2b).

### *CsZFP6* is the candidate gene for *CsGLF1*

The 111.6 kb SNP marker-defined region on chromosome 5 contains 10 annotated genes (Chinese Long cucumber v3 reference genome; http://cucurbitgenomics.org/) (Table S3, see online supplementary material). To refine the list of candidates, we performed RNA-seq on fruit pericarps and flesh of the parental lines. The transcriptomic results revealed that of the 10 candidate genes, four were extremely low expressed in pericarp: *CsaV3_5g028900*, *CsaV3_5g028940*, *CsaV3_5g028950*, and *CsaV3_5g028960*. Considering that glossy-fruit is a fruit epidermal related trait, these four genes were not pursued further. Of the six remaining candidates, *CsaV3_5g028870*, a predicted homolog of *ZFP6*, was completely absent in the DDX as revealed by PCR amplification and sanger sequencing (Fig. S2, see online supplementary material), which was consistent with biparental whole-genome resequencing data (Fig. S3, see online supplementary material), while no nucleotide variation was detected in other candidates. Besides, *CsaV3_5g028870* was preferentially expressed in pericarp versus flesh and differentially expressed between DDX and 93–46 (Fig. 2c). Collectively, these analyses indicated that the loss of *CsaV3_5g028870* (designated as *CsZFP6* hereinafter) results in the glossy-fruit phenotype of DDX.

### CsZFP6 positively regulates cuticular wax biosynthesis

**Figure 3:**
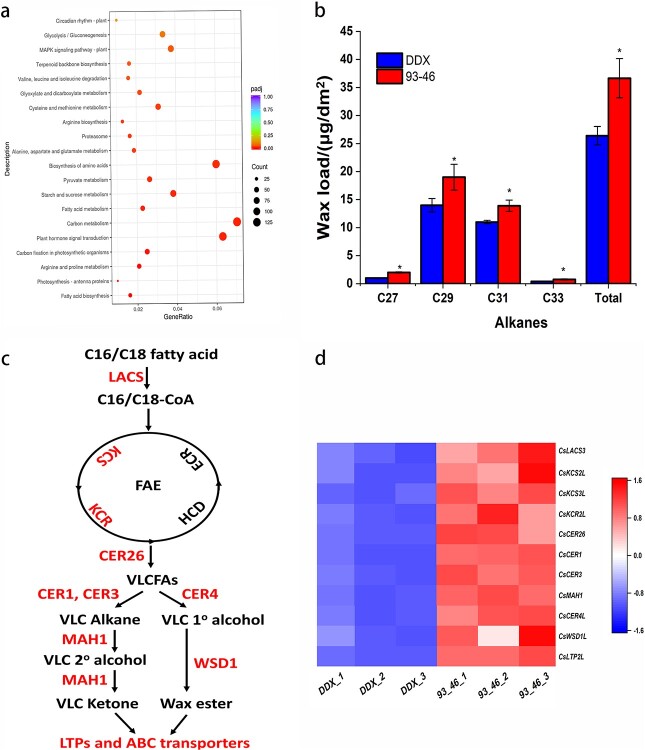
CsZFP6 positively regulates cuticular wax biosynthesis. **a** The enriched KEGG pathways of DEGs between DDX and 93–46. The *x*-axis represents the gene ratio, and *y*-axis represents the KEGG pathway. The size of each dot indicates the gene number that was annotated in this pathway. The dot color represents the padj-value. **b** GC–MS quantification (expressed as μg/dm^2^) of pericarp cuticular wax from the DDX and 93–46. Biological triplicates were averaged and statistically analysed using a Student’s *t* test (^*^P < 0.05). The error bars indicate the standard error of mean. **c** Schematic diagram of a simplified biosynthesis pathway of cuticular wax. DEGs are marked with red. **d** Heat map of wax-related DEGs in DDX and 93–46. Three biological replicates were performed and the colored bar on the right of the map represents normalized FPKM value.

Previous findings showed that ZFP6 is a C2H2 transcription factor which regulates trichome initiation in inflorescence organs [23–25]. Here, our mapping results implied a novel role of this protein in affecting cucumber glossiness. In order to investigate the regulatory mechanisms of CsZFP6 in the fruit glossiness regulation, transcriptome analysis was carried out to identify putative target genes of CsZFP6. Differentially expressed genes (DEGs) were identified between DDX and 93–46 by selecting the FPKM values with the following criteria (Padj <0.05, |log_2_foldchange| > 1). As a result, a total of 3377 DEGs were identified, including 1632 upregulated genes and 1745 downregulated genes in the 93–46 (Table S4, see online supplementary material). Kyoto Encyclopedia of Genes and Genomes (KEGG) enrichment analysis revealed that these DEGs were mainly enriched in the signaling transduction, amino acid biosynthesis, carbohydrate metabolism, terpenoid biosynthesis, and fatty acid biosynthesis pathway (Fig. 3a).

**Figure 4:**
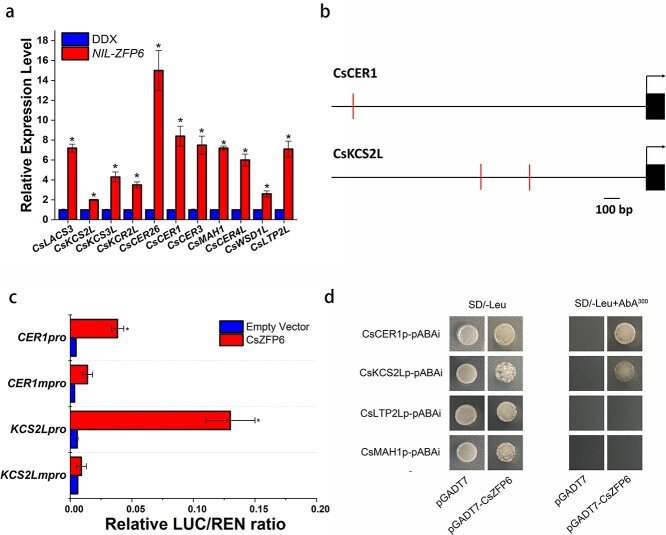
CsZFP6 positively regulates cuticular wax biosynthesis. **a** qRT-PCR of cuticular wax-related gene expression. Biological triplicates were averaged and statistically analysed using a Student’s *t* test (^*^P < 0.05). The error bars indicate the standard error of mean. **b** Diagrams of the *CsCER1* and *CsKCS2L* promoters. Positions of the A[AG/CT]CNAC motif are indicated. The bent arrow indicates translational start sites. **c** Tobacco transient expression assays showing that CsZFP6 transactivates cuticular wax biosynthetic genes through A[AG/CT]CNAC motif. Biological triplicates were averaged and statistically analysed using a Student’s *t* test (^*^P < 0.05). The error bars indicate the standard error of mean. **d** Yeast one hybrid analysis of the interaction between CsZFP6 and the promoters of *CsCER1* and *CsKCS2L*. Physical interaction was determined on SD/−Leu medium in the presence of 300 ng/mL AbA. The empty pGADT7 vector was used as negative control.

Fatty acid has been shown to be the precursor of cuticular wax biosynthesis. Glossy phenotype due to wax deficiency has also been reported in *Arabidopsis* [9], *Brassica* crops [4, 6], maize [5], peach [8], sorghum [26], and rice [27]. Measurements of cuticular wax content and composition by gas chromatography showed that the wax load was significantly elevated in 93–46 (Fig. 3b; Table S5, see online supplementary material), which is in accordance with our transcriptomic data showing that the transcript levels of main wax-related genes were increased (Fig. 3c and d). To verify the above result in a common genetic background, we constructed a *CsZFP6* Near-Isogenic Line (NIL) and measured the expression levels of 11 main wax-related genes by qRT-PCR. Consistently, these genes were shown to be greatly induced in the *CsZFP6* NIL (Fig. 4a).

### CsZFP6 trans-activates wax biosynthetic genes

Typically, C2H2-type ZFP transcription factors bind to the A[AG/CT]CNAC motif of gene promoters [24]. We analysed the promoter sequences of those 11 main wax-related genes and found that promoters of *CsCER1*, *CsKCS2-Like* (*CsKCS2L*), *CsLTP2-Like* (*CsLTP2L*) and *CsMAH1* contain the A[AG/CT]CNAC motif (Fig. 4b; Fig. S4, see online supplementary material). It is likely that these genes are regulated by CsZFP6. To test this, we employed a dual-luciferase reporter approach. In this experiment, the wax-related gene promoter-driven firefly luciferase reporter and 35S promoter-driven Renilla luciferase were constructed in the same plasmid and transiently expressed in *N. benthamiana* leaves. We monitored the LUC:REN ratio, and as expected, CsZFP6 was able to transactivate these four wax-related genes (Fig. 4c; Fig. S5, see online supplementary material).

We then conducted yeast one hybrid assay to test whether CsZFP6 binds to the promoter of these four genes. Promoter-fused pAbAi bait vector and pGADT7-CsZFP6 prey vector were cotransformed into the Y1H gold yeast strain. As shown in Fig. 4d, yeast cells containing pAbAi-CsCER1 or pAbAi-CsKCS2L were able to grow on 300 ng/mL aureobasidin A (AbA) SD/−Leu media, indicating that ZFP6 associates with upstream regions of *CsCER1* and *CsKCS2L.* On the contrary, yeast cells containing pAbAi-CsMAH1 or pAbAi-LTP2L failed to grow. To further investigate the underlying mechanism, *CsCER1mpro:LUC*, in which the putative CsZFP6 binding site was mutated, was coexpressed with *35Spro:CsZFP6* in *N. benthamiana* leaves. As shown in Fig. 4c, the activation effect of *35Spro:CsZFP6* was largely attenuated. Likewise, coexpression of *35Spro:CsZFP6* and *CsKCS2Lmpro:LUC* failed to elevate LUC:REN ratio (Fig. 4c). Thus, it is likely that CsZFP6 regulates *CsCER1* and *CsKCS2L* expression through its binding motif.

## Discussion

Remarkable fruit glossiness diversity characterizes cultivated cucumbers. In cucumber, glossy-fruit is an important visual quality trait that affects consumer acceptance. It has been revealed that the cucumber glossy-fruit trait was determined by a single recessive gene *d*^3^. However, F_1_ plants derived from the cross between DDX (glossy European greenhouse-type inbred line) and JY35 (glossy north China-type inbred line) displayed dull fruits (Fig. S1, see online supplementary material), indicating the regulatory mechanism on cucumber glossiness is complicated. To clone the underlying fruit glossy genes, a RIL population was constructed. In the present study, we identified a glossy fruit gene on Chr5 by map-based cloning, using a F_2_ segregation population derived from the cross between a dull RIL 93–46 and DDX. The combined evidence from genomic sequence and gene expression analysis support *CsZFP6* being the candidate gene, and loss of *CsZFP6* due to the 4895 bp deletion between the promoter and the coding region confers cucumber fruits glossy. Gene expression analysis revealed that *CsZFP6* is preferentially expressed in pericarp (Fig. 2c; Fig. S6, see online supplementary material), and consistently loss of *CsZFP6* does not lead to glossiness change on leaves (Fig. S7, see online supplementary material). A previous study in *Arabidopsis* has shown that ZFP6 is a C2H2-type zinc finger protein which controls trichome initiation in inflorescence organs [25]. C2H2-type zinc finger proteins were reported to regulate multiple plant biological processes such as epidermal cell differentiation, root hair elongation, shoot development, and stress response [28–30]. Here, we demonstrate a novel role of C2H2-type zinc finger protein in controlling fruit glossiness.

It is noteworthy that *CsZFP6* has been previously referred to as the *Tuberculate Fruit* (*Tu*) gene responsible for the cucumber warty fruit trait [23, 31]. The cucumber wart consists of spines (trichome on fruit) and underlying tubercules. In this work, we present that CsZFP6 not only determines tubercule formation, but also participates in the regulation of fruit glossiness, which explains why cucumber cultivars in Europe generally feature glossy fruit with no or few spines and warts [32]. Consistently, our collected glossy-fruit cucumber accessions from Eurasia exhibit the non-warty trait. As both warty and glossy fruit are important visual quality traits that greatly influence market value, *CsZFP6* will be a putative useful target for marker-assisted selection or genome-editing to improve cucumber appearance in breeding. Previous linkage analysis demonstrated that *Micro-trichome tubercule* (*Mict*) and *Trichome-less* (*Tril*) are epistatic to *CsZFP6* [23, 32], both of which encode HD-ZIP transcription factors [33, 34]. Recently, it has been revealed that cucumber lines absent of *Mict* gene which belongs to the HD-ZIP I subfamily also displayed glossy-fruit [35]. Although it has not yet been reported that whether *Tril*, which encodes a HD-ZIP IV transcription factor functions in the regulation of cucumber glossiness, its homolog *Woolly* (*Wo*) in tomato has been shown to affect glossiness by modulating wax biosynthesis [36, 37]. Thus, we speculate that these three core transcription factors in controlling cucumber warty fruit trait are also involved in regulating fruit glossiness. Nonetheless, the underlying mechanism remains largely unexplored.

An EAR motif (DLHLSLA sequence) is present at the C terminus of CsZFP6, indicating that this transcription factor may function as transcriptional repressors in gene regulatory networks. However, our present findings revealed that CsZFP6 act as a transcriptional activator in regulating wax biosynthesis. Further experiments need to be performed to verify whether CsZFP6 is able to directly bind to the promoter of wax biosynthetic gene *CsCER1* and *CsKCS2L in vivo*. The glossy phenotype due to wax deficiency has also been reported in other plants previously such as *Arabidopsis* [9], broccoli [4], peach [8], maize [5], and rice [27]. Therefore, we speculated that the glossy-fruit phenotype of DDX is also due to decreased wax content. Consistent with this speculation, we found that the wax load as well as main wax biosynthetic gene expression level were reduced in DDX.

## Materials and methods

### Plant materials and growth conditions

Two cucumber inbred lines, DDX (European greenhouse-type) and JY35 (North China-type) were used in this study. A RIL population containing 81 individual lines was derived by single-seed descent from a cross between DDX and JY35. All plants were self-fertilized to the F_8_ generation. To clone the *CsGLF1*, we generated an F_2_ population derived from the cross between a dull-fruit RIL 93–46 and DDX. To perform the gene expression analysis in a common genetic background, we constructed a *CsZFP6* Near-Isogenic Line (NIL). The detailed scheme for NIL development is described in Fig. S8 (see online supplementary material). Cucumber seedlings were grown in in 50-plug trays containing sterilized peat until the second true leaf stage and then transferred to the greenhouse of the Yangzhou University (Yangchow, China). The fruit glossiness was observed visually and scored three times by different people at 7 d post-pollination (dpp).

### Gloss level measurement

Gloss levels of 7-dpp cucumber fruits were measured by a XA6 Curve Gloss Meter (JND, Shanghai, China). Each measurement was repeated five times.

### Map-based cloning of *CsGLF1*

Glossy inbred line DDX was crossed to 93–46, a RIL with dull-fruit derived from DDX and JY35. In the resulting F_2_ population, 76 polymorphic InDel markers between the parents were used for preliminary mapping by bulked segregant analysis (BSA) [4]. Briefly, two extreme DNA pools (15 glossy F_2_ individuals for the glossy pool and 15 dull F_2_ individuals for the dull pool) were created to detect InDel markers linked to *CsGLF1*. Then, polymorphic markers between the two DNA pools and their nearby InDel markers were applied to all F_2_ individuals to confirm the linkage and narrow the location of the target gene. For fine-mapping, SNP markers within the preliminary mapping region were designed according to whole genome re-sequencing data [38]. Recombinant individuals were screened within an enlarged F_2_ population of 500 plants. All primers used for mapping were listed in Table S2 (see online supplementary material).

### RNA-seq analysis

Total RNA was isolated from the 7-dpp cucumber fruits with three biological replicates. cDNA libraries were sequenced using NovaSeq 6000 platform (Illumina, San Diego, CA, US). Raw reads were processed by removing adapter sequences and low-quality reads. The paired-end clean reads were mapped to the Chinese Long cucumber v3 reference genome. Gene expression levels were estimated using the fragments per kilobase of transcript per million mapped reads (FPKM) method. Differential expression analysis was carried out using the DESeq2 R package (1.20.0). Genes with an adjusted *P*-value <0.05 and |log_2_foldchange| > 1 were assigned as differentially expressed. KEGG pathway enrichment analyses were performed using the clusterProfiler R package.

### Cuticular waxes loads and compositions detection by GC–MS

The assay was performed as described previously with minor modification [39]. Cuticular waxes were extracted from 7-dpp cucumber fruits in chloroform for 5 min at room temperature. Tetracosane was added to the extracted chloroform solvent as internal standards. Extracts were dried under a stream of N_2_ gas and converted into trimethylsilyl derivatives by heating at 90°C for 30 min in *N,O*-bis(trimethylsilyl)trifluoroacetamide):trimethylchlorosilane (99:1). Samples were analyzed by ISQ QD GC–MS (Thermo, Waltham, MA, US).

### qRT-PCR assay

qRT-PCR was performed on iQ™ 5 multicolour Real-Time PCR detection system (Bio-Rad, Hercules, CA, US). The expression of *CsACTIN* (CsaV3_2G018090) was used as an internal control and the expression of other genes was calculated using the 2^-△△CT^ method. Primers used are listed in Table S6 (see online supplementary material). Data were analysed from three independent sets of biological replicates.

### Dual-luciferase assay

The assay was carried out in tobacco leaves as described previously [35]. Briefly, *CsZFP6* coding sequence was cloned into pGreenII_0029_62-SK vector driven by the 35S promoter. The promoters of *CsMAH1*(−2027 to −1 bp), *CsKCS2L*(−1442 to −2 bp), *CsLTP2L*(−2109 to −1 bp), and *CsCER1*(−2429 to −14 bp) were cloned into pGreenII _0800-LUC to drive the firefly luciferase reporter gene. To generate mutated CsKCS2L promoter in which two putative ZFP6 binding sites were replaced with TTTTTTT, PCR was performed using pGreenII_0800-LUC_ CsKCS2Lpro plasmid as the template and three pairs of primers: LUC-KCS2Lpro-F/KZBS1-R, KZBS1-F/KZBS2-R, and KZBS2-F/LUC-KCS2Lpro-R. The resulting three DNA fragments were fused by an additional PCR reaction using the primers LUC-KCS2Lpro-F and LUC-KCS2Lpro-R. The fused product was also cloned into pGreenII _0800-LUC. Likewise, to generate mutated CsCER1 promoter in which the ACTCAAC motif was replaced with TTTTTTT, PCR was performed using two pairs of primers: LUC-CER1pro-F/CZBS-R and CZBS-F/LUC-CER1pro-R. The resulting two DNA fragments were fused by an additional PCR reaction using the primers LUC-CER1pro-F and LUC-CER1pro-R. The fused product was also cloned into the pGreenII _0800-LUC. Vectors were transformed into *Agrobacterium tumefaciens* GV3101. Agrobacterium-mediated infiltration of *Nicotiana benthamiana* leaves was performed as described [40]. After 72 h, tobacco leave discs were collected and luciferase activities were quantified using dual luciferase reporter assay kit (Vazyme, Nanjing, China). The primers used are listed in Table S6 (see online supplementary material).

### Yeast one-hybrid assay

Yeast one-hybrid assay was performed using the Matchmaker Gold Yeast One-hybrid System (Clotech, Clontech, Mountain View, CA, US). The promoter sequences of *CsMAH1*(−2027 to −1 bp), *CsKCS2L*(−1442 to −2 bp), *CsLTP2L*(−2109 to −1 bp), and *CsCER1*(−2429 to −14 bp) were inserted into the pAbAi, and the coding sequence of *CsZFP6* was cloned into pGADT7. The recombinant pAbAi vectors were linearized and integrated into the Y1H Gold yeast strain genome to test the promoter autoactivation on SD/-Ura medium according to the system manual. Subsequently, pGADT7-CsZFP6 plasmid was transformed into bait yeast strains, and the positive yeast strains were screened on SD/−Leu medium with 300 ng/mL AbA at 28°C. All primers used are listed in Table S6 (see online supplementary material).

## Accession numbers

All genes used in this article can be found in the CuGenDB under the following accession numbers: *CsCER4L* (CsaV3_1G014400), *CsKCS3L* (CsaV3_1G031130), *CsCER3* (CsaV3_1G029420), *CsCER26* (CsaV3_2G011770), *CsKCS2L* (CsaV3_3G012730), *CsLACS3* (CsaV3_3G026550), *CsLTP2L* (CsaV3_4G027160), *CsZFP6* (CsaV3_5G028870), *CsKCR2L* (CsaV3_5G030680), *CsCER1* (CsaV3_6G006560), *CsMAH1* (CsaV3_6G042360), *CsWSD1L* (CsaV3_7G008480). The raw sequence data of this study were deposited in the sequence read archive of the National Center for Biotechnology Information under accession number PRJNA808486.

## Acknowledgements

This work was supported by grants from the National Natural Science Foundation of China (32272718), Jiangsu Key Research and Development Program (BE2022339), Jiangsu Agricultural Science and Technology Independent Innovation Fund Project (CX(20)3165), the Key Project For Jiangsu Agricultural New Variety Innovation (PZCZ201720), the Jiangsu Provincial Entrepreneurship and Innovation Doctor Program, the Yangzhou City’s Green and Golden Phoenix Program, Modern Agriculture Foundation of Yangzhou (YZ2020036), and the Promoting Project for Open Competition Mechanism to Select the Best Candidates of Jiangsu Seed Industry (JBGS[2021] 018).

## Author contributions

X.C. and C.C. designed the study. Y.Y., C.C., Y.P.W., and Y.R.W. performed the research. C.C., Y.P.W., Y.R.W., and H.J. analysed the data. Y.Y., C.C., and X.C. wrote the manuscript. All authors read and approved the final manuscript.

## Data availability statement

The raw sequence data of this study were deposited in the sequence read archive of the National Center for Biotechnology Information under accession number PRJNA808486.

## Conflicts of interest

The authors declare no competing interests.

## Supplementary data

Supplementary data is available at *Horticulture Research* online.

## Supplementary Material

Web_Material_uhac237
