# Supplementary material for: Cucumber glossy fruit 1 (CsGLF1) encodes the zinc finger protein 6 that regulates fruit glossiness by enhancing cuticular wax biosynthesis
Source: Hortic Res. 2022 Feb 21;10(1):uhac237. doi: 10.1093/hr/uhac237 (PMC9832831; doi:10.1093/hr/uhac237)
Supplement: Web_Material_uhac237 [file web_material_uhac237.zip › Fig S2 20220702.pdf]

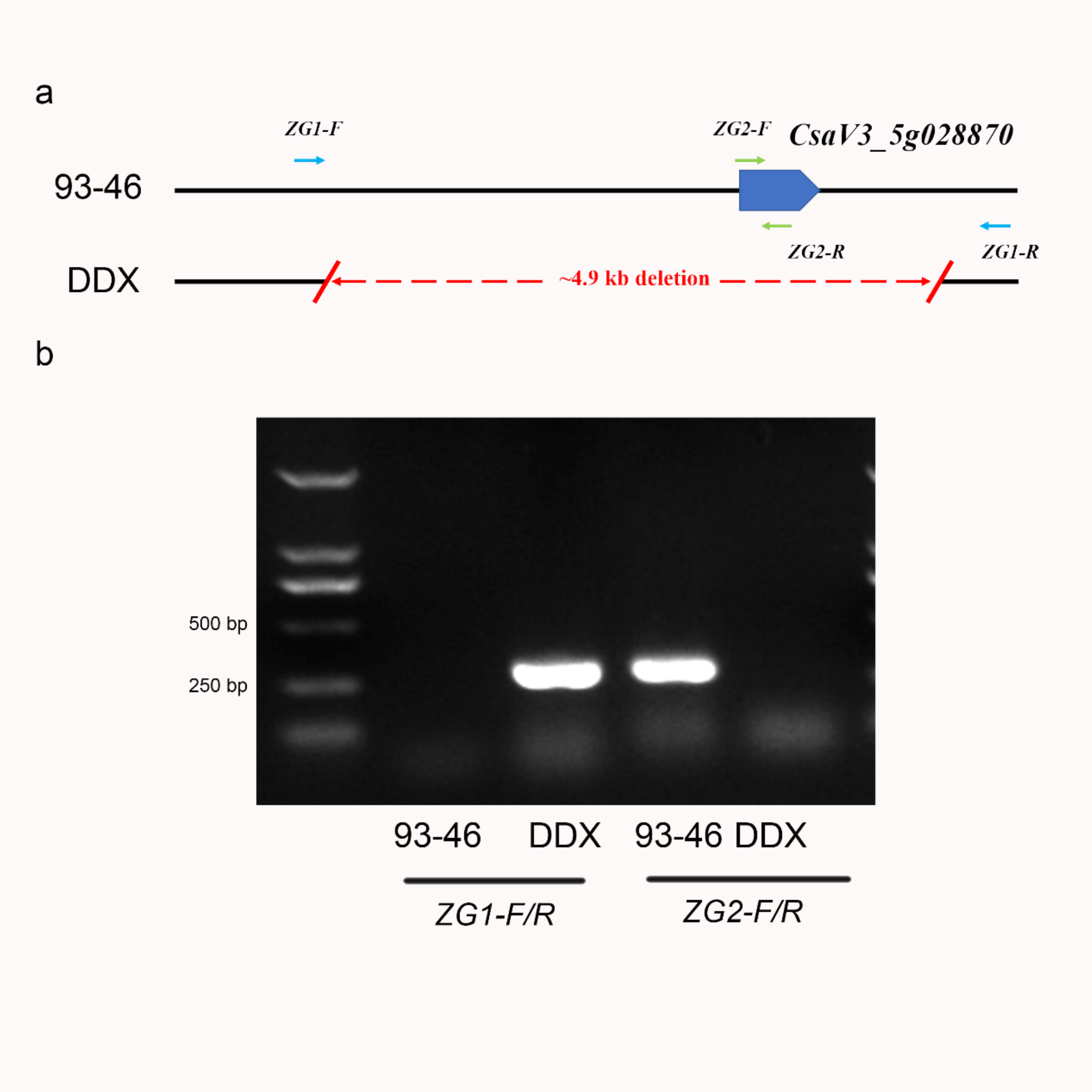

Fig. S2 Genomic structure analysis at *Csgpl1*. a Genomic sequence difference between DDX and 93-46 at the *Csgpl1* locus. **b** PCR amplification using primers *ZG1-F/R* and *ZG2-F/R*.
