# Supplementary material for: Cucumber glossy fruit 1 (CsGLF1) encodes the zinc finger protein 6 that regulates fruit glossiness by enhancing cuticular wax biosynthesis
Source: Hortic Res. 2022 Feb 21;10(1):uhac237. doi: 10.1093/hr/uhac237 (PMC9832831; doi:10.1093/hr/uhac237)
Supplement: Web_Material_uhac237 [file web_material_uhac237.zip › Fig S3 A 4895â_%bp deletion was detected in DDX..pdf]

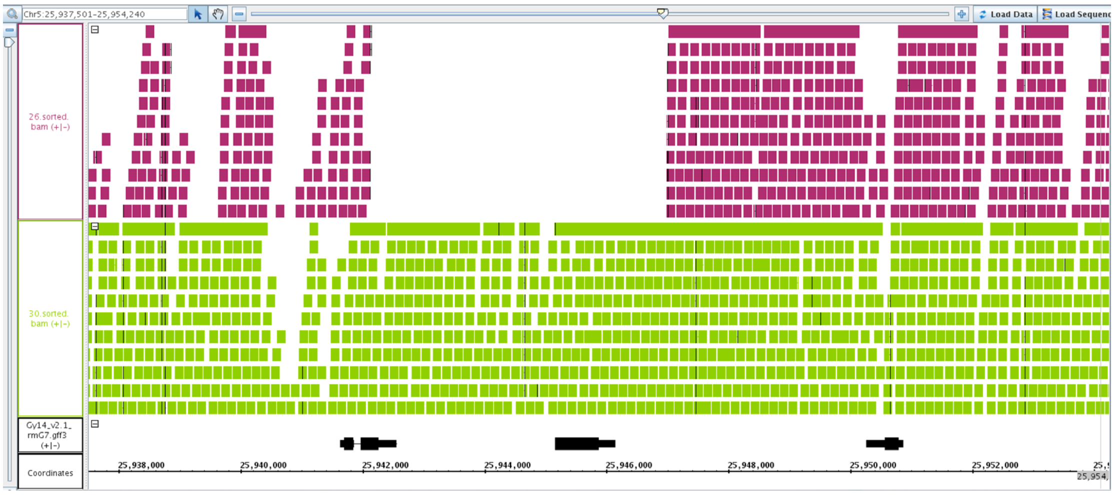

Fig. S3 A 4895 bp deletion was detected in DDX. Whole genome resequencing data of DDX and JY35 are visualized by loading bam files into Integrated Genome Browser (version 9.0.2). Each 150bp long read were represent by a rectangle (magenta for DDX and light green for JY35). Raw reads were trimmed with trim galore (Ver 0.6.8) and aligned to reference genome (http://cucurbitgenomics.org/ftp/genome/cucumber/Gy14/v2/) by bowtie2(4.8.5), SAM files were converted into BAM files by using samtools(1.15.1)
