# Supplementary material for: Cucumber glossy fruit 1 (CsGLF1) encodes the zinc finger protein 6 that regulates fruit glossiness by enhancing cuticular wax biosynthesis
Source: Hortic Res. 2022 Feb 21;10(1):uhac237. doi: 10.1093/hr/uhac237 (PMC9832831; doi:10.1093/hr/uhac237)
Supplement: Web_Material_uhac237 [file web_material_uhac237.zip › Fig S4(ZFP6 binding site).pdf]

Fig. S4 *CsCER1*, *CsKCS2L*, *CsLTP2L* and *CsMAH1* contain C2H2-type ZFP binding element in their promoters. C2H2-type ZFP binding element is highlighted in purple. ZFP binding motif is taken from: Sun, L.L. et al. GLABROUS INFLORESCENCE STEMS3 (GIS3) regulates trichome initiation and development in *Arabidopsis*. *N. Phytol.* **206,** 220-230 (2015).

CsCER1 (CsaV3_6G006560)

-2000 CATAGTGATAGAAAGAAACAAATAATACCAAAAATAAATCATGGAAAAA
-1950 CGAAATTAAATATGTATGATCTAATTATAAAAGAGGGAGAAGAAACATA
-1900 GCCATAGATTGTTGGTGGGATATGATGGAAGTCGAGTTATTATCATTAT
-1850 CTTTATCATAAAATCTATTTTTATGAAAATATACTGAATTGTGTCGATT
-1800 GGTGTGTGAAATTATTTAAATGACAATTTTTAAAAATATCACAATCGA
-1750 TAGAATTAACATGCTACTATCTTGATCTACCAAAATTTGATTGTTATGA
-1700 TATTTTGCTATATATTTTGATTCATTTGTTAAGTTTGAAAGTAATAATT
-1650 ATATATAAAGATGGAGTCTCGAACACATAGGTATGGAAGACATATTAAT
-1600 GTATCATAATATAATGGGCATAATTGTTAGAGGAATCAACTTCAAGACA
-1550 GCAGTGAGACAAAAAAAAAAAGAGTATTTAATATAAGTTTTTTAATTTA
-1500 ATAATAAAACAATAAGAATAAAATAAATTTTTAATAACAAAAGATGCAT
-1450 GTAGGTTTTTCTTGAATTAAGTTTGAAAAAGTTGAGAGAAAAAGAAAAG
-1400 ATCTCATAGTCACATAGACCTAAAAGCACTTTTCTTCAAGGACATTCAT
-1350 TATGCATATATCTCCAAACATATATGTATATATGTATAATCTCATGAAT
-1300 CCAATTTTCCATATATACCTAGTAAATTAAAATAAAAGAAAACATCTGC
-1250 CACATTTTAATGTTTATTGGTGTGCAAGTTGCAAAGACATATGAGTTGC
-1200 AACATAACACCTAGTTTTTTTTTTTTTTCTCTTTTAATTTCACAATTGT
-1150 TGGTGTAAGAATTACATTATTGCTGTACAAATAGGTAAATAATAATAAC
-1100 AAAATAAAAAACCTCTCTAATAATTTTCACTATCTTCTAATAATTTTCA
-1050 CTATCTTCAATTTGTTTGGAATTTAATTTCAACCTGACATGTGTATCTT
-1000 ACAAAATCCACCGAATGTTACTATTTTATTTTATAGGCAGTTTGGCAAA
-950 GTATGAAATATTTACATAACCACTAAACATTTGTATATTCAATACATAC
-900 AACAAGGTGGATGGAATGTAAAGAAACTATACACAAAGAACTGTAGACG
-850 CTAAGAATAATGAGTAGGGAATTTATTCTTATTTTAATAAGTTGTATTA
-800 GTTTAGAAAAATAATACTTTTAAATTTAAGAAAAATAAATCAAAATATT
-750 TACAAAATAATATAGAAAATTTTTATATAATCTATAGATTGAGAGACTC
-700 TTATAGAAGTCTATCATGTCTATCATTACTATATTACAAAATTTTACTA
-650 TAATATTTTGTAAGTGATTTATGGGAATGTTGATGGTAAAGATGGCTTT
-600 GTTAGATAAATATTTAAAAACATTAACTTATATTATTAATGTCTCTCCC
-550 CGTATAAATTCAAAACCTTTGTTCTAATAATGTAATAGATATGGATGAA
-500 AGAACAAGTGCGAGAGCTATATACCTAATTATGTATATGCTACGTACTC
-450 ACCAACCCATAAAAGTAATAAAAAGAAAAAGAAAGGAGAGTATACTGTA
-400 TTCCTCCAACTGTCAAATTACCTAAATCATTAATTTTCCACCAAAGTAG
-350 ATAAATTTTAAAAACTTGTTATCAAAAGAGTTTAAATCAAAATATTTTT
-300 TCTAGTCAATTCAAACACTGCACTGTTCTGAATGAGTAAAGAATATGGA
-250 TGCAAACAAGTAGCCCAAGTGTGTTTTCTTAATGGTTTAACTTCAAAAG
-200 TGCAAACAAGTAGCCCAAGTGTGTTTTCTTAATGGTTTAACTTCAAAAG

-150	GGCATGCAGTTGTAATTTCCTCATATGTTGGTGGCTAATATGGTGAACT
-100	TTCACTTTCACTTTCACCAAGCTAAGTGAGACTCTCTCTATATATAATC
-50	TTGTGACACCCATTGCCATGAAATATTGTAACCTTCTAAAACAATAACA

CsKCS2L (CsaV3_3G012730)

-1200	TTCTCTATAATCATATCATTCAATAATATTATAATAAGCGAAAATTTTA
-1150	GGAATATAATAACTAAATATATAGTGACATTTAAAGAAAAATCAATCTA
-1100	AAATAATTAGAAAAATAAAATAAATGCAATACAAATATAAAATAAACTC
-1050	TACTAAGCTTTTGAGAAATATTCTCGTAATGATTCACAAAAACTTCCTA
-1000	AAAAAAGTAGAAAAAACCGGGAGGAAGAAAAAAAAACCTCAATTGTCAT
-950	AAAATATGTAAACCACTCCATTATTATTCCATCAAGGAGTTAACATAAT
-900	TTTACATTCTTTTTAAAAGTTTAAAATTTAAAACAAACGTTTTAGAAAA
-850	TTGGAGGATGAAAATAAAAACATATACAAAGGTTGAAGATCAAAATAAA
-800	ATTTAAACATAATATCAACCTTTCTGTCGCTTCTATTTAGGTTGATTTT
-750	ATTGTTCTTCAAGCTAACATACATTTGAGATATTTGGAGTGATAATCTT
-700	AAATCTAATACAGTTTTAGTGTATAAATTAAGACAAATGATGGCCAATT
-650	TAGCTAAGCACCGTAAAAACAAGATAAAATCTCTAATTTGAAGATCACT
-600	TAAATCTCTTATTTAGGATCCACTTATAATTTAATCATGTGATACATAT
-550	GTATTGATTATTATGTTAGTGAGGTTTATGTTGGTGGCGTTTGTAAGAG
-500	TTGAATTAAAATTGAACGTAGTTATTTTTCAACAATTATTAAGATGTTT
-450	CTAGCTGGGTGGATGAACATAAACTTCCAAACTACGATTATAATTAATA
-400	TGTGTATGTGTATGTGTATGTATGAAGATGGCAGATTAGATAGTTTATG
-350	AATATTTGAATGAAAATTTCCGACATGAAACATCAGCGTAAAAGTAAAT
-300	GAAGAAGTAAGACAGAGTTTTCCGGGTTGGCAAATTGAATGCCCCCCCG
-250	CCTTCCCCTTCCCTTCACAATTAAAAGTTCGTTGGGGTGGTTGTATCAG
-200	TATCCTCTCCCTCCCCCCTCCCCTTCTCTTCCTCTTTTCTCTTCACACT
-150	TCTCATCATCCCCTCCCACCCCTCCTCTCTTTTATAAAACCCCCCCTCT
-100	TATCTCATTTCAATCCCATTCTTCTTCTTCTTCTTCTTCTGCTCTT
-50	TCTCATCATCAAAAAACTAAACTAAACAAACAAACAAACAAACAACAAA

CsLTP2L (CsaV3_4G027160)

-1100	TCTTTCCTAAAAATTAGTAATTGATTATTTGTTATTAATGTTTTATAAA
-1050	CTACTTTTTTCTCAAAAAAAAAAAAAAAACAAAATAGGTTTATAAACCA
-1000	CTCAATAAAACAAAATTATACTCTAAAGATTTAAGTTGAGTGGAAAGTT
-950	GGTTTTTGTGTTTTCAAATTCGATATATAATCTTTAATATTTGGAAAAA
-900	TGGTTTATTTTCTTTTATTATAGTAACTAACAAAAAAAAAAAAATTGACT
-850	TGACATTCCTACTAATTAAGAAATTGAGAGTTTAAATTTAATACAACTT
-800	TAAATATTAGAGTGTGAAATTTTTCTTTTTTTAAAAAGCTTAAAAACTA
-750	AAATTAATAGACCTTACAAAAATTATAGACTTTTTAAGAATAATATAAA
-700	AAAAACTAAATTAAAAATGTTAAAAATTATTTACACTCTCAAATTTATA
-650	ACCATTTAATTTTTTTAAATTTAATATATATAATTACAATTGAGTTACT
-600	ATATTTTAAAATGCATAAAATTTCAGTCTTTTAACATAAAAAAATCTTT
-550	ATAAAAAACTATTTTTCATTAGTCTTAATAGTTTATGATCATTTTAATA
-500	GATCTATATATATAAATATTTTTATTCTATTGTGTATTTTTTATTCAAT

-450 ATTCGATACACAATAGACTAAGAGTACTTTTAGATTGCTCTTTTTAAAG
-400 TTAAAACTGTTTTTATAAAAGAAAATTTACTTTTAAAATACACTTCAAA
-350 AGTTTTTTTTTTGAGTGGTTTTCAGACCGATAAAAGTGATTCAAAATGA
-300 TTAATATTACAAATTCAAGAGGTGATTAACGAAAAAACCTTGGATCTAA
-250 AACATAGAAAAAACTTACACTACACCATTACTCATAAAGCAAAGCATTT
-200 AACATACAAACGTTAGTAGGTGGCGTTTTAAAACTATCACCAACCAATC
-150 GAAAGCTGCATTTATCACCGCCAAACCTACCAATCCCATCCCCCATTCA
-100 TTTTCCATTCTATTTAAACTCCCCAAGCTCTCCCACTTCACACTCAAAC
-50 TCATCTTACAAACTCCTTCAAATTTCTATTTACTTTTCGTAAACACATC

CsMAH1 (CsaV3_6G042360)
-2000 CTTAATTAATTCATAAAATCATATTCAATTAGTATAATTAAAATATGAG
-1950 AGGTAAAGGACAACATTGTCATCTACCTAATTACATAAGCTACTTCTAA
-1900 AGGAAGGTCAATAATAATGGAAAAAAAAATTCTCCTCGTCCAAGCTTTT
-1850 ATATATACTACTAGTAAACAACATGTGCATTAAAATCACACACGAACAA
-1800 AATTATAGTATTAATTTAACTTTATAAAATTGAAACCGTTATTATAATA
-1750 TTTTCATATTTATATCATTTTTATATTCACATCGTAATGAGTGAAAAAT
-1700 GAATTTATTTCGTAAGGAGAAAATGTTGTCATCTTTTGAATGTATCTTT
-1650 GAATTTGATTTGTTTGCACAATTCAAATTTTATTTCTTAACATCTTGAA
-1600 AAACAATGTTAACATATATCATTGTGTAATATCAATTATGAAAGATTGA
-1550 AAAAATACATTATGACCCTAATAACATAGAGCATTAACTAATATCAGTT
-1500 ATGGATCAAAACATTTTTTTTCAAATAAGGAGATGAAAAGTTAACAAAG
-1450 TGTAACTTCTATTTTGTTGAATGTAAAAATGATCGCAAACATTAAATCT
-1400 ATGTGAATGATAAAATTGTTATAATGAAAAGGGTAAAACATCCCCAAAA
-1350 TCATATTTCTTTTGAAATTATGAAATTATAAATGTGAAATCATAATTTT
-1300 TTTATAATGATTTTATAGGTGAAAAAATAAAGAAAAGACTTTAATATTT
-1250 CTTGTTTTACACATTTTATTATATTTATTTATACTTAATAAATTTCAAC
-1200 GATATTTACATTTTTTTTTAAATTAAAGCATAAGTCACTCAACCATTTC
-1150 ATTAATAAAATAGTTTTAATAAAAAAAATTAAATAAAGAAAAAAACATG
-1100 AGCAAGATTTTCATGGTTACATTAAAAAAAGATTTCAATATTAAATGA
-1050 TTTACATATGTTGGTGGTTCAAGTTTCCCCAACTTTTGTTGTGTACTAA
-1000 AAAGAAACTTATAAATGAATGAATAAACAAAAAAAAAAATAAAACAAAA
-950 AAAAAAAGAATAGAAAAACATGATTTGATTTCTTCTTCATCTTTAACAT
-900 TTTGTCGTGACTTGTGAATATGATAAAAAAAAAAATATTTATAACAAGAT
-850 TTGGAAATAAGAGATATGAAAAGTTAAAAGAGATAGGAAATGTGAATAG
-800 TTGACGAGAGATAGAAGATTAAAGGAGAGAAGAGAATATGAATGAATTT
-750 GGATAAATTTAAATGACATAATTAATTGGAGTGATTGTTTCTTTAAAAC
-700 AAAGATTATAACAAGGAGATGAAAAGTTAAAAAAATCATTCTCACTTTA
-650 TAATGAAAATGATTCAAATATTAAATCTATATAAATGATAAAATTGTCA
-600 CTAAATCAAAATTGAAAAGTTAACTAATGAAAGGGTAAAATGGAGCTAA
-550 AAAAATTTCTCACACTTCCATCTTTTATCTATAGTATACATAAATATAG
-500 ATTTAATTATTAGTTTATTTCAATTTAATTATTCTCATATACTACATAT
-450 GTTTGTAATTATAGTTATTTATTATGGTGTTTTTCATGTAACTTATAAT
-400 ATTATTTATTTGTAACATAACTCTCCAAATTCCCTTTTTCTCATATTTT

-350 TATCTTTTAGTTTCAAATTTTTACCATAACTCTCAAAATAATTTATCTT
-300 TAATACAAAAACTACAAAATCTCAGATATATATGTGTTTGGAATTTTTT
-250 CTAAACCATTCCATTCCTTTGGAGACATGAAATCTATTGATTTTATCAC
-200 TTGTAGTAACTTCTCATTTAAGAAGATATTAAAGTTAGGTTTTCAATCC
-150 ATCAGTCATCTAGTCCAAAGTCATCCCCAACAGAGGTAAACCCATGAAC
-100 AGTGGAGATTGGAGACCATGTAGTGTTCAACTATATATATATCCACACT
-50 CATCATACAAAAAAAGTTTAATTGAGTGTTTTTGTAGAGATCAGCTCCA
