# Supplementary material for: Cucumber glossy fruit 1 (CsGLF1) encodes the zinc finger protein 6 that regulates fruit glossiness by enhancing cuticular wax biosynthesis
Source: Hortic Res. 2022 Feb 21;10(1):uhac237. doi: 10.1093/hr/uhac237 (PMC9832831; doi:10.1093/hr/uhac237)
Supplement: Web_Material_uhac237 [file web_material_uhac237.zip › Fig S5.pdf]

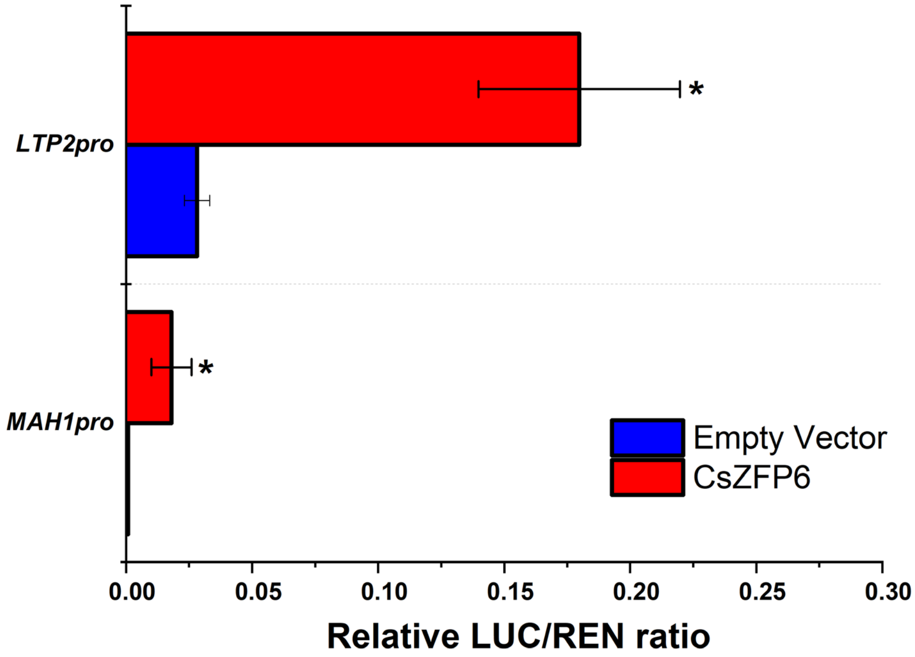

Fig. S5 Tobacco transient expression assays showing that CsZFP6 transactivates the expression of *LTP2L* and *MAH1*. Biological triplicates were averaged and statistically analyzed using a student's t test (*$p < 0.05$). The error bars indicate the standard error of mean.
