# Supplementary material for: Cucumber glossy fruit 1 (CsGLF1) encodes the zinc finger protein 6 that regulates fruit glossiness by enhancing cuticular wax biosynthesis
Source: Hortic Res. 2022 Feb 21;10(1):uhac237. doi: 10.1093/hr/uhac237 (PMC9832831; doi:10.1093/hr/uhac237)
Supplement: Web_Material_uhac237 [file web_material_uhac237.zip › Fig S6 Relative gene expression of CsZFP6 in 93-46 organs..pdf]

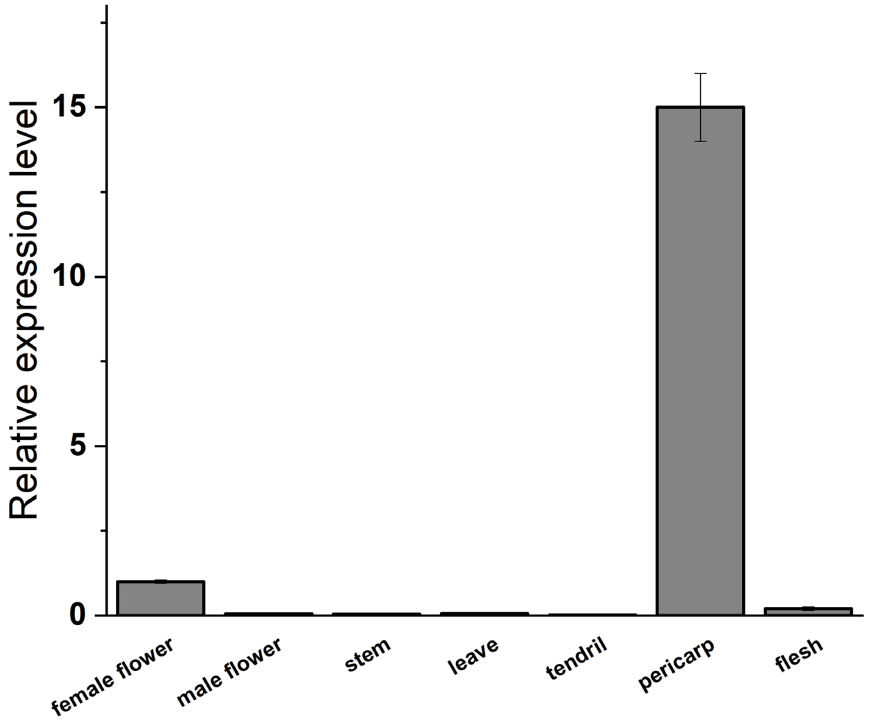

Fig. S6 Relative gene expression of *CsZFP6* in 93-46 organs. Each data point represents the mean of three independent biological replicates (mean±SD). Values are shown compared with expression level in female flower.
