# Supplementary material for: Cucumber glossy fruit 1 (CsGLF1) encodes the zinc finger protein 6 that regulates fruit glossiness by enhancing cuticular wax biosynthesis
Source: Hortic Res. 2022 Feb 21;10(1):uhac237. doi: 10.1093/hr/uhac237 (PMC9832831; doi:10.1093/hr/uhac237)
Supplement: Web_Material_uhac237 [file web_material_uhac237.zip › Table S1.docx]

Table S1 Genetic analysis of the glossy-pericarp trait in the F_1_ and F_2_ populations derived from DDX and 93-46

| Cross | Population | Total | No. of Dull plants | No. of glossy plants | Expected ratio | χ^2^ value^1^ |
| --- | --- | --- | --- | --- | --- | --- |
| DDX×93-46 | F_1_ | 30 | 30 |  |  |  |
|  | F_2_ | 182 | 136 | 46 | 3:1 | 0.0563 |

^1^χ^2^(0.05,1)=3.84
