# Supplementary material for: Cucumber glossy fruit 1 (CsGLF1) encodes the zinc finger protein 6 that regulates fruit glossiness by enhancing cuticular wax biosynthesis
Source: Hortic Res. 2022 Feb 21;10(1):uhac237. doi: 10.1093/hr/uhac237 (PMC9832831; doi:10.1093/hr/uhac237)
Supplement: Web_Material_uhac237 [file web_material_uhac237.zip › Table S3.docx]

Table S3 Annotation of 10 candidate genes within the 111.6 kb region

| No. | Gene ID | Gene annotation |
| --- | --- | --- |
| 1 | *CsaV3_5g028870* | Zinc finger protein |
| 2 | *CsaV3_5g028880* | Phosphoenol pyruvate carboxylase protein |
| 3 | *CsaV3_5g028890* | Nudix hydrolase 1-like |
| 4 | *CsaV3_5g028900* | Protein MIZU-KUSSEI 1-like |
| 5 | *CsaV3_5g028910* | Protein IDA |
| 6 | *CsaV3_5g028920* | Protein CROWDED NUCLEIS |
| 7 | *CsaV3_5g028930* | Protein decapping 5-like |
| 8 | *CsaV3_5g028940* | Clavaminate synthase-like protein |
| 9 | *CsaV3_5g028950* | Protein CHUP1 |
| 10 | *CsaV3_5g028960* | Unknown protein |
