# Supplementary material for: Cucumber glossy fruit 1 (CsGLF1) encodes the zinc finger protein 6 that regulates fruit glossiness by enhancing cuticular wax biosynthesis
Source: Hortic Res. 2022 Feb 21;10(1):uhac237. doi: 10.1093/hr/uhac237 (PMC9832831; doi:10.1093/hr/uhac237)
Supplement: Web_Material_uhac237 [file web_material_uhac237.zip › Table S5.docx]

Table S5 Content of cuticular wax compositions (μg.dm^-2^) in DDX and 93-46.

| Wax composition | | DDX | 93-46 |
| --- | --- | --- | --- |
| VLCFAs | C22 | 1.1±0.1 | 1.6±0.2* |
|  | C24 | 2.2±0.2 | 2.0±0.1 |
|  | Subtotal | 3.2±0.4 | 3.5±0.2 |
| Alkanes | C27 | 1.1±0.1 | 2.2±0.1* |
|  | C29 | 14.2±1.1 | 19.8±2.3* |
|  | C31 | 11.0±0.3 | 13.9±1.2* |
|  | C33 | 0.4±0.1 | 0.8±0.1* |
|  | Subtotal | 26.7±1.5 | 36.7±3.5* |
| 2-alcohols | C29 | 1.1±0.2 | 1.4±0.3 |
| Total | | 31.0±1.9 | 41.6±4.0* |

Biological triplicates were averaged and statistically analyzed using a student’s t test (**p* < 0.05).
