# Supplementary figures and images for: Cucumber glossy fruit 1 (CsGLF1) encodes the zinc finger protein 6 that regulates fruit glossiness by enhancing cuticular wax biosynthesis
Source: Hortic Res. 2022 Feb 21;10(1):uhac237. doi: 10.1093/hr/uhac237 (PMC9832831; doi:10.1093/hr/uhac237)

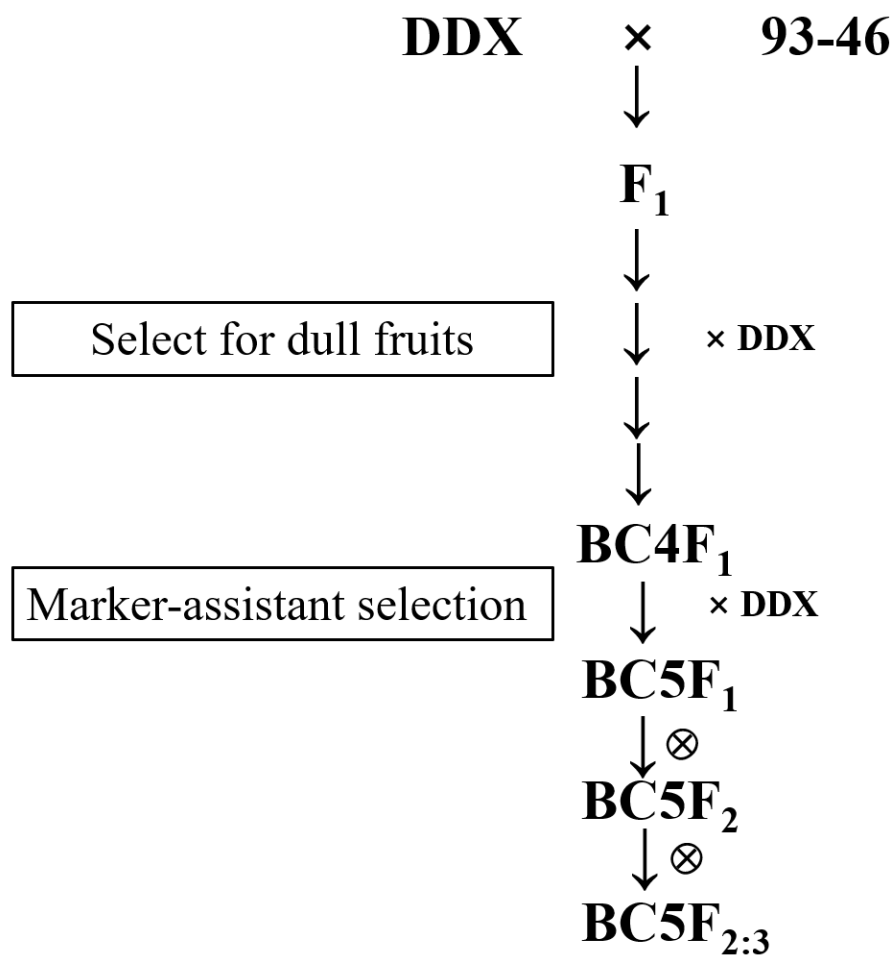

**Fig. S8 Breeding scheme for development of *NIL-ZFP6*.**

Supplement: Web_Material_uhac237 [file web_material_uhac237.zip › Fig S8 NIL-ZFP6 development.pdf]
